# 

**Published:** 1858-04

**Authors:** 


### AMERICAN DENTAL CONVENTION

*Subjects for discussion at the next Annual Meeting.*

"The business committee appointed at the convention at Boston, would respectfully present the following topics for discussion at the next convention to be held in Cincinnati, commencing the first Tuesday of August, 1858.

1. The best means of *securing* and preserving good teeth.
2. *Treatment* of Exposed Nerves.
3. Mechanical Dentistry.
4. Filling Teeth.
5. Miscellaneous.

J. Taft, C. W. Spalding, C. A. Harris, E. Townsend, B. Lord, *Committee.*"

JNO. T. TOLAND.

### CORRESPONDENTS.

We earnestly solicit every individual who receives a copy of the "Reporter," to lend us their aid and assistance, not only in giving circulation to our enterprise, but in endeavoring to make it what it should be, a medium of intelligence between the different members of the profession.

A few words from each one will fill the work every three months with such a *variety* of *practical* matter as will be astonishing. It is no excuse to say, you don't know of any thing of sufficient *importance*; every business, and every profession, and every science, is made up of trifles in themselves, which lead to investigation and the accumulation and development of great principles. If you have nothing to impart, you can ask questions which will draw out communications from others.

We do not expect nor want long, fine spun theories, with an eloquent display of words, elegantly turned periods! but plain, practical information, short and spicy. Come, gentlemen, lend a helping hand, for as you give, so shall you receive.

### SUBSCRIBERS.

We expect *every dentist* who receives this number, not only to become a subscriber himself, but also act as agent in procuring subscriptions among the physicians, druggists, and the intelligent portion of the public, in fact all who feel any interest in the advancement of dental science, and there is not one who should not feel interested in a science so intimately connected with the comfort, convenience, of eating and speaking, in personal appearance, and above all in the influence exercised over the general health.

We do not ask you to make it take the place of any other Journal, there are but few published, and there is no danger of learning too much if you take them all.

As remarked in the introduction, we can afford to publish cheap just in proportion to the liberality or extent of subscriptions. In an edition of 500, the principal expense is the type-setting. In an edition of 5000, the principal expense is *paper* and *press-work.*

I do not expect any profit in this, if the *subscriptions* pay the *expense,* I will be satisfied.

I have, therefore, resolved on a first edition of 3000, expecting to increase it at least 1000 with each additional number. Our weekly newspapers circulate from 50,000 to 100,000, and there is no reason why the Reporter should not have at least as many.

I think the profession know me well enough to take in trust my assurance, that it shall not be a mere bundle of wordy trash, and I have confidence enough in them to believe that they will second my efforts in making it valuable, and in giving it a wide circulation.

I have, therefore, resolved to put the price so low, that none will feel the expense, as follows:

| | |
|---|---|
| One copy one year,........................... | 25 cents. |
| Five copies, one year to one address,............. | $ 1,00 |
| Thirty copies, one year, to one address,.......... | 5,00 |
| One Hundred copies, one year, to one address,..... | 15,00 |

Those who will get up clubs, can thus realize a handsome profit for themselves.

*All subscriptions must be* CASH IN ADVANCE, as at this low price we can not afford to keep *book accounts,* nor incur the expense of sending out *collectors.*

Many of my best friends have urged me against this course, on the ground that the very low price would condemn it as worthless, and consequently I would lose money. But I have *confidence* in the *cause* and in the *profession.*

### APOLOGY.

We must apologize to our friends and the profession, for the delay of this number, as well as for the limited amount of original matter.

There are always more or less unexpected and necessary delays, in getting out the first issue, which will not occur when the wheels are in motion and well greased, which they will be in future.

The amount of space occupied by the report of the proceedings of the Miss. Valley Association of Dental Surgeons, which we presume will be esteemed as interesting as any thing we could publish; as also, the necessary introductory remarks and explanations, have necessarily crowded out much interesting original matter, which will appear in the next number.

In future, we hope to have no occasion for explanations, and we intend to have the enterprise move along like "clock-work." In this, we expect the aid of every one who receives this, to lend a helping hand. Send in your communications early, "*Short and Sweet*" is our doctrine.

We do not confine our remarks to those who receive this number, because we know there are a great many whose names we have not on our list, it is not our intention to slight any one. And we will be pleased to receive the names and residence of every dentist, physician, and druggist, in the United States, and also receive communications from them for publication.

We design this to be a perfectly FREE AND INDEPENDENT JOURNAL, and will be pleased to publish articles, (free from personalities), on any subject of *interest* to the profession, the more *spicy* the better.

### ADVERTISERS.

To advertisers, the "*Reporter*" offers superior advantages to any other DENTAL JOURNAL, its circulation being much larger than any other, and from three to five times as great as some.

We print for the present number 3000 copies, and expect to *increase* with each issue.

The circulation extends to *every state* in thé Union, but more especially *west* of the Allegheny mountains, where it goes to *every town* of any importance. It goes into the hands of the most INTELLIGENT and LIBERAL portion of the community, viz: *dentists, physicians*, and *druggists*. Being a *book of reference*— it remains on the table in the offices of subscribers, where it is seen and frequeutly examined by the *numerous visitors*, who are waiting for operations or advice.

TERMS OF ADVERTISING.

| | |
|---|---|
| One page for one year, | $40,00 |
| Half a page " | 25,00 |
| One fourth of a page, one year, | 15,00 |
| One eighth page one year, | 10,00 |

*Special* advertisements by *contract.* Advertisements to be *paid* in *advance.*

Those who wish to avail themselves of the facilities of *extensive* advertising at *small expense* as above, will please forward their copy, accompanied a *remittance* to *pay* for the amount of *space* they wish to occupy, according to the above terms, this will save the time and trouble of correspondence, and insure *prompt insertion.*

*Advertisements* for the next issue should be sent as early as the 15*th of June.*

### NEW DENTAL LATHE.

In the advertising columns of the April number of Dental News Letter is an illustration of a " New Dental Lathe," which for neatness, beauty of appearance, and compactness, excels anything we have seen.

We have just received some for sale. They run light and steady. Price, $13.50. With three chucks and corundum wheels, $15.00.

JNO. T. TOLAND,
38 *West Fourth street, Cincinnati.*

### EXTRACTING FORCEPS WITH SHIFTING BEAKS,

As illustrated in News Letter. Price $20 per set. Furnished to order by

JNO. T. TOLAND,
38 *West Fourth st., Cincinnati, O.*

### CURRENCY BANKABLE IN CINCINNATI.

| | |
|---|---|
| OHIO—Par, except Seneca County Bank | 60 |
| Union Bank of Sandusky | 5 |
| City Bank of Columbus | 5 |
| " Cincinnati | 30 |
| Canal Bank, Cleveland | no sale |
| KENTUCKY—Par, except People's Bank | 1 per cent. dis |
| INDIANA.—State Bank and Bank of the State | par |
| Free Banks, specie paying, | 1 per cent. dis |
| LOUISIANA | Par |
| N. Y. CITY AND STATE | Par |
| CONNECTICUT—Par, except Bank of Hartford, Co | 2½ per ct. dis |
| " Litchfield, " | 2½ " dis |
| " North America | 10 " dis |
| Hatters' Bank | 1½ " dis |
| Litchfield Bank | 1½ " dis |
| Uncas " | 2½ " dis |
| Woodbury " | 2½ " dis |
| Wooster " | 2½ " dis |
| DELAWARE | Par |
| DISTRICT OF COLUMBIA.—All 3 per cent., except Farmers & Merchants' Bank, and Corporation of Washington, Georgetown and Alexandria Notes, for which there is no sale. | |
| GEORGIA.—Savannah Banks, 2½ per cent. All others, 5 to 20. | |
| ILLINOIS—Specie Paying | 2 per ct. dis |
| MARYLAND.—Baltimore City Banks | Par |
| " Country Banks, 1 per cent., except Cumberland Savings Bank | 5 per ct. dis |
| MAINE | Par |
| MASSACHUSETTS | Par |
| MICHIGAN | 3 to 5 per ct. dis |
| MISSOURI | 1½ per ct. dis |
| NEW HAMPSHIRE | Par |
| NEW JERSEY—Par, except America Bank, Trenton | 50 per ct. dis |
| NORTH CAROLINA | 3½ per ct. dis |
| NOVA SCOTIA | 10 to 15 dis. |
| PENNSYLVANIA.—Philadelphia Banks par, except Bank of Penn. | |
| " Country Banks | 1½ to 5 per ct. dis |
| except Erie Bank | 10 per cent. dis |
| Warren Co. Bank | 10 " " |
| Tioga Co. Bank | no sale |
| RHODE ISLAND | Par |
| SOUTH CAROLINA | 1½ dis |
| TENNESSEE.—Nashville Banks | 3¼ per ct. dis |
| Country Banks | 5 to 50 per ct. dis |
| VERMONT | Par |
| VIRGINIA | 3½ per cent. dis |
| except Wheeling Banks | 1 per cent. dis |
| " Branches | 2 per cent. dis |
| WISCONSIN | 1½ to 2 per ct. dis |

EXCHANGE ON NEW YORK.

BUYING—½ to ¾ prem. SELLING—¾ to 1 per cent. prem.

### CINCINNATI PRODUCE MARKET.

THURSDAY, April 15th, 1858.

FLOUR—Active. Sales of 3100 bbls. at $3 60@$3.65 for superfine, and $3.75@$3.85 for extra. Receipts small.

WHISKY.—Prices declining. Sales 1800 bbls. @ 16½c.

IRON.—Sales of 600 tons Tenn. Pig @ $27.

PROVISIONS.—Mess Pork in demand @ $16.75. Holders ask $17. Sales of 400 bbls. at former prices. No demand for Shoulders. Sides are wanted, and prices firm @ 9¼ cts. Lard is in good demand, with sales of 1200 bbls. @ 10c.

CANDLES.—Star have advanced to 19c.

TALLOW.—Sales of 100 bbls. @ 10c on 60 days.

OIL.—Advancing ; sales of 41 bbls. Linseed @ 70.

GROCERIES.—Molasses higher, with sales of 250 bls. @ 35c. Sugar firm ; sales 150 hhds. at 7¼ @7¾c. Coffee unchanged ; sales 950 bags prime at 11@12c.

WHEAT.—Market firm. Sales 400 bush. White at 70c ; 300 bush. good Red at 72c, and 200 bush. good white @ 83c.

CORN.—Market firm at 35c.

BARLEY.—Declining. Sales 1000 bush. spring Barley Malt @ 60c.

RYE.—Scarce and firm at 55c.

OATS.—In fair demand at 32@33c.

BALED HAY.—Demand good for prime at $13@$13.50. Common grades dull at $7@$10.

POTATOES.—Dull, but prices firm. Sales 1000 bush. Meshannocks at 25 cts, and 200 bush. Pink Eyes at 20c.

### THE ECLECTIC COLLEGE OF MEDICINE.

### Cincinnati, O.

Two sessions are held annually, the first commencing early in October, and the second early in February. Further information may be obtained by addressing the *Dean* of the Faculty.

### FACULTY.

J. F. JUDGE, M. D., 295 Fifth St.
*Professor of Chemistry and Pharmacy.*
T. E. ST. JOHN, M. D., 141 Seventh St., (corner of Elm.)
*Professor of Anatomy and Physiology.*
A. JACKSON HOWE, M. D., 141 Sixth St.
*Professor of Surgery.*
C. H. CLEAVELAND, M. D., 139 Seventh St.
*Professor of Materia Medica and Therapeutics.*
WM. SHERWOOD, M. D., 243 Court St.
*Professor of Medical Practice and Pathology.*
JOHN KING, M. D., 113 Smith St.
*Professor of Obstetrics and Diseases of Women and Children.*
J. R. BUCHANAN, M. D.,
*Emeritus Professor of Cerebral Physiology and Institutes of Medicine.*

### COMMENDATIONS.

We can cheerfully recommend this College to all young men who desire to attend a course of Lectures.—*The Messenger, Bloomington, Mo.*

It numbers among its Professors some of the ablest in this country.—*Yates Co. Chronicle, N. Y.*

It is one of the most efficient in its preparation of students for Western Practice, of any in the United States.—*Courier, Indianapolis, Ind.*

The Faculty of this Institution have a high reputation for ability.—*Intelligencer, Lancaster, Pa.*

This is a highly popular Institution.—*Courier, Prairie Du Chien, Wis.*

This College has gained the reputation of being one of the best in the U. S., and affords unrivalled facilities for instruction.—*Journal, Muskegon, Mich.*

It stands high at home, and wherever known abroad.—*Ashland Kentuckian.*

This is the best Institution of the kind in the West.—*Telegraph, Wash. Ind.*

This Institution affords to the student rare facilities for acquiring a thoroughly practical knowledge of medicine.—*Leader, Lexington, Mich.*

It has connected with it some of the most intellectual men of the age.
*Union Democrat, Columbia, Mo.*

This Institution bears a high reputation as thorough and scientific.
*Reveille, St. Charles, Mo.*

This Medical College is one of the best in the United States.
*The Courier, Opelousas, La.*

The Eclectic College, at Cincinnati, offers fine advantages to students who have an inclination to pursue the study of medicine.—*Pennsylvanian, York, Pa.*

The Institution is as capable of imparting instruction to students as any of our Eastern Schools.—*Church Advocate, Harrisburgh, Pa.*

This school is spoken of in very flattering terms by many. To young men desiring a medical education it presents great inducements.—*The Democrat, Conneaut, O.*

---

### The College Journal of Medical Science,

A Monthly Magazine of 48 pages, conducted by the Faculty of The Eclectic College of Medicine, is published at One Dollar a Year, payable in advance. The volume of the *Journal* commences with the year. Communications for subscription, or for specimen numbers, should be directed to

Dr. C. H. CLEAVELAND, Publisher,
139 Seventh Street, Cincinnati, O.

WRIGHTSON & CO., PRINT., 167 WALNUT STREET, CIN.

